# Bone Loss after Allogeneic Haematopoietic Stem Cell Transplantation: A Pilot Study on the Use of Zoledronic Acid

**DOI:** 10.1155/2012/858590

**Published:** 2012-04-10

**Authors:** Andreas Hausmann, Wolfgang Hill, Hans Joachim Stemmler, Georg Ledderose, Andrea Baur-Melnyk, Susanne Fritsch, Johanna Ullmann, Hans-Jochem Kolb, Sandra Geiger, Johanna Tischer

**Affiliations:** ^1^Medical Department III, Ludwig-Maximilians University of Munich, Campus Großhadern, 81377 Munich, Germany; ^2^Department of Radiology, Ludwig-Maximilians University of Munich, Campus Großhadern, 81377 Munich, Germany

## Abstract

*Purpose*. Bone loss is a common phenomenon following allogeneic haematopoietic stem cell transplantation (allo-HSCT). The study aimed on tolerance and efficacy of zoledronic acid (ZA) in patients after allo-HSCT. *Methods.* 40 patients' with osteoporosis or osteopenia were recruited on this phase II study. ZA was given at a dose of 4 mg IV every 3 months for 2 years (yrs). BMD was determined by dual-energy X-ray absorptiometry (LS lumbar spine, FH femur hip). Patients were evaluated for deoxypyridinoline (Dpd) and calcium excretion by longitudinal measurements. *Results*. 36 patients who had received at least 3 doses of ZA were evaluable. 26 patients had at least two BMD measurements since baseline (BMD group). Among these patients, BMD increased from 0.97 ± 0.15 to 1.10 ± 0.18 g/cm² (LS baseline—2 yrs, Δ+11.6 ± 6.0%, *P* < 0.001) and from 0.82 ± 0.10 to 0.91 ± 0.10 g/cm^²^ (FH baseline—2 yrs, Δ+7.5 ± 7.0%, *P* < 0.001). Factors associated with an increase in BMD were younger age, female donor sex, and immunosuppression with CSA/MTX. *Conclusion*. ZA was generally well tolerated; it increases BMD and reduces Dpd excretion significantly in patients with bone loss after allo-HSCT.

## 1. Introduction

Bone loss is recognized as one of the most frequent complications in long-term survivors after allogeneic stem cell transplantation [1–4]. Data on the temporal sequence of bone loss in these patients are sparse [4–6]. Contrary to patients who underwent transplantation of a solid organ, these patients are exposed to numerous factors that may affect the skeletal system: induction and/or consolidation chemotherapy of the malignant hematologic disease, high-dose chemotherapy, malignancy-related changes in bone structure, total body irradiation (TBI) and consecutive hypogonadism, graft-versus-host disease (GvHD), immunosuppressive treatments including corticosteroids and cyclosporine A (CSA), and immobilisation [1, 7–9].

Prospective studies of bone loss in patients after allo-HSCT are sparse, and cross-sectional studies revealed conflicting data. A positive correlation of bone loss was found for corticosteroid and CSA use, baseline BMD, and loss of muscle mass [5]. 

Oral bisphosphonates are widely used for treating osteoporosis and have been shown to improve BMD and decrease the rate of fractures in various patient populations [10]. However, the use of the oral formula is compromised by poor gastrointestinal tolerance. About half of patients after HSCT suffer from GvHD, with the gastrointestinal tract being one of the most frequent targets [11, 12]. Intravenous bisphosphonates have proven to be effective in patients with hemato-oncological malignancies. This includes a clinical benefit in reduction of bone pain, and reduction and/or delay of skeletal complications [13]. ZA has been evaluated in some small studies for efficacy and safety in patients after allo-HSCT; however, the majority of these studies covered a relatively short period of 12 months posttransplantation [14–17].

The present study aimed at investigating the 12- and 24-month effect of ZA administration on lumbar and femoral BMD and bone turnover markers. Moreover, these data were correlated to clinical features. Finally, we identified risk factors which were associated with significant bone loss in this setting.

## 2. Patients and Methods

The study protocol was approved by the institutional ethics committee. Informed consent was given by the patient prior to study entry.

Forty patients after allo-HSCT were recruited on this prospective, monocentric phase II study between 2001 and 2006 at the Ludwig-Maximilians University Hospital of Munich.

The general aim of the study was to evaluate efficacy and tolerance of a 24-month interval therapy with ZA in patients with osteopenia/osteoporosis after allo-HSCT.

### 2.1. Inclusion and Exclusion Criteria

Eligibility criteria were as follows: signed informed consent, allogeneic HSCT within 4 years until inclusion, age ≥18 years, osteoporosis (T-score ≤−2.5 SD) or osteopenia (T-score −1.0 to −2.4), KPS ≥70%.

Exclusion criteria were relapsed underlying malignancy, and serum creatinine >1.5 mg/dL, history of tooth extraction or surgery of the jaw during the last six months, prior bisphosphonate treatment, or women with hypogonadism without adequate hormone replacement therapy.

### 2.2. Primary and Secondary Objectives

The main variable was the mean percentage change of BMD of the lumbar spine (L1–L4) compared to baseline. Patients were screened for BMD by dual energy X-ray absorptiometry (DXA-Lunar prodigy). Secondary endpoints included BMD of total femur hip, parameters of bone modulation in serum and urine, for example, deoxypyridinoline (Dpd), and calcium excretion in urine by longitudinal measurements.

### 2.3. Therapy Performance

ZA was given at a dose of 4 mg IV every 3 months for 2 years (yrs) in combination with calcium (>500 mg/d), vitamin D_3_ (>400 IE/d), and, in case of hypogonadism, hormone-replacement therapy.

### 2.4. Statistics

According to recent trials in patients with reduced BMD after allo-HSCT, the mean percentage annual bone increment will be estimated at 7.2% by a SEM of 0.49% and a standard deviation of 2.7%, respectively. To detect these changes by means of a paired *t*-test (two-sided) and an error probability of *α* = 0.05 and a power of 80% (*β* error 0.2), a number of at least 4 patients was necessary. Due to planned statistical analyses in subgroups and a high drop-out rate of 20% because of high morbidity and mortality after allo-HSCT, an overall number of 40, including 25 evaluable patients, have been estimated. Statistical analysis was performed with SPSS Version 18 for Windows. Differences between baseline and 1-year and 2-year measurements were analyzed by paired *t*-test. A *P* value <0.05 was considered to be statistically significant.

## 3. Results

### 3.1. Patient Characteristics

**Figure 1:**
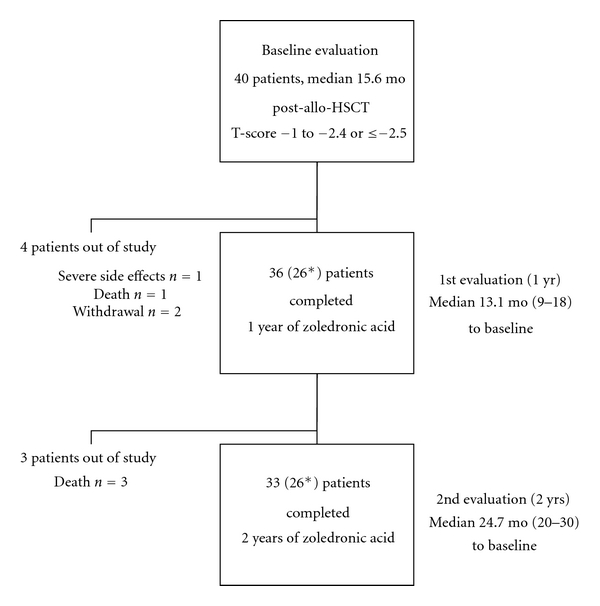
Study design and flowchart. *BMD and Dpd measurements = BMD group (by DXA).

Forty patients fulfilled the inclusion criteria. Baseline examination was median 15.6 months after allo-HSCT. 36 patients (24 male, 12 female; median age 43.8 yrs) who had received at least 3 doses of ZA were evaluable. None of them had fractures. 26 out of those patients had at least two BMD measurements since baseline (BMD group) (Figure 1). During the post-allo-HSCT period, patients had received different immunosuppressants: 24 patients had received cyclosporine A (CSA) and methothrexate (MTX), whereas one-third (12 patients) had received CSA and mycophenolate mofetil (MMF). Moreover, patients had received considerable doses of corticosteroids (prednisolone) with cumulative doses of 11.1 g prior to study entry and additional 5.4 g during the investigational phase. The duration of corticosteroid treatment was 152 days prior to study entry, and 628 days during the study. The majority (28 out of 36 patients) developed clinical signs of acute GvHD, which was mild in 5 patients (grade 1 or 2) and severe (grade 3 and 4) in 23 patients. Chronic GvHD was observed in 26 patients and was graded to a limited stage in 4 and to an extensive stage in 22 patients, respectively.

Detailed information of baseline characteristics and type of allo-HSCT is given in Table 1.

### 3.2. BMD Measurements, T-Score

**Figure 2:**
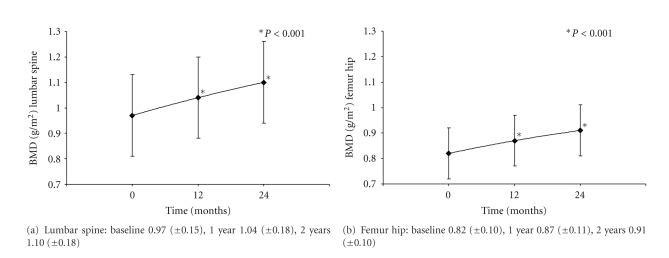
BMD (mean ± SD; g/m²) of lumbar spine (a) and femur hip (b) after treatment with zoledronic acid.

The baseline examination of BMD was median 15.6 months after allo-HSCT. 26 out of 36 patients had at least two BMD measurements since baseline (BMD group). Among these patients, BMD (mean ± SD) of the lumbar spine significantly increased from 0.97 ± 0.15 to 1.10 ± 0.18 g/cm². BMD of the femur hip increased from 0.82 ± 0.10 to 0.91 ± 0.10 g/cm² (Figure 2). The relative increment of BMD of the lumbar spine within 2 years (Δ BMD LS baseline—2 yrs) was 11.6 ± 6.0% (*P* < 0.001). The corresponding relative increment of the femur hip (Δ BMD FH baseline—2 yrs) was 7.5 ± 7.0% (*P* < 0.001).

**Figure 3:**
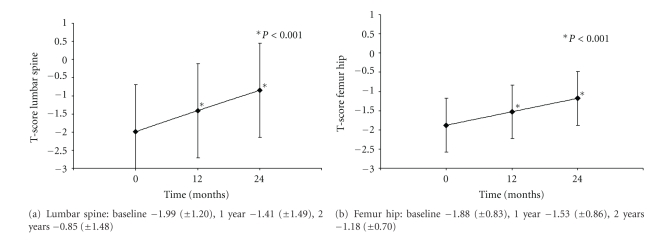
T-score (mean ± SD) of lumbar spine (a) and femur hip (b) after treatment with zoledronic acid.

T-scores (mean ± SD) of lumbar spine and femur hip increased significantly compared to baseline (*P* < 0.001). In detail, the T-score of the lumbar spine was −1.99 (±1.20) at baseline and increased to −1.41 (±1.49) at 1 year and to −0.85 (±1.48) at 2 years. The corresponding T-scores of femur hip was −1.88 (±0.83) at baseline, −1.53 (±0.86) at 1 year, and −1.18 (±0.70) at 2 years (Figure 3).

The increase of BMD (mean ± SD) was significantly pronounced in younger patients (<45 yrs) than in older patients (BMD FH: Δ+ 8.9 ± 5.8% versus + 2.9 ± 2.9% at 1 yr, *P*  0.007; Δ+ 12.0 ± 8.1% versus 3.4 ± 3.9% at 2 yrs, *P*  0.02). Another factor which was associated with a significant increase in BMD was female donor gender. The percentage increment of BMD of the femur hip at 1 year was 7.6 ± 5.4% versus 2.9 ± 4.3% (female versus male donor, *P*  0.04) and 10.2 ± 7.8% versus 4.4 ± 5.3% (female versus male donor, *P*  0.05), at 2 years, respectively. Finally, immunosuppression with CSA/MTX was associated with a significant increase in BMD. The percentage increment of BMD of the femur hip at 1 year was 7.1 ± 5.1% versus 2.2 ± 3.2% (CSA/MTX versus CSA/MMF, *P*  0.01) and 9.3 ± 8.0% versus 3.9 ± 2.7% (CSA/MTX versus CSA/MMF, *P*  0.02), at 2 years, respectively.

Other analyzed factors as corticosteroid therapy prior to or during the study, baseline BMD, and underlying malignancy prior to allo-HSCT were not significantly associated with changes of BMD.

Detailed information is given in Table 2.

### 3.3. Metabolic Parameters and Safety

36 patients had received at least 3 doses of ZA according to the study protocol (median 5 doses, range 3–8). Deoxypyridinoline (Dpd, nmol/nmol creatinine) decreased from 6.2 ± 4.6 to 3.6 ± 3.2 after 1 yr (*P*  0.009) and 3.2 ± 1.4 after 2 yrs (*P*  0.04). There was no significant change of any other evaluated parameter (Table 3).

In general, ZA was well tolerated. One out of 40 patients was excluded after the first dose due to severe myalgia. Mild flu-like syndromes, bone pain, chest pain, or headache have been observed in 6 patients. None of the patients developed hypocalcaemia or increased creatinine serum levels (Table 3). None of the patients developed osteonecrosis of the jaw or fractures during the study interval.

## 4. Discussion

Transplantation of hematopoietic stem cells is a widely accepted treatment option for various hematologic diseases. Despite constantly increasing survival rates, allo-HSCT is still associated with a considerably high morbidity and mortality. Therefore, long-term survivors after allo-HSCT are confronted with new problems if they develop chronic osteopenia or osteoporosis [5].

Factors which are associated with bone loss are age, hypogonadism, presence of GvHD and subsequent use of corticosteroids, and other immunosuppressants [2, 3, 8, 17].

Data from prospective studies of bone loss in patients after allo-HSCT are sparse. Moreover, these studies revealed conflicting data. Studies with an observational period of 3, respectively, 4 years, demonstrate a rapid bone loss within the first 6 months [18]. In the most recent study provided by Schulte and Beelen, the nadir of BMD was at 6 months for lumbar spine and at 24 months for femoral neck [5]. In the univariate analysis they found that only few factors contribute for the risk of rapid bone loss, namely, cumulative steroid dose and average steroid dose per day, average duration of exposure to CSA, and negative changes in muscle mass and high baseline BMD [5]. In the multivariate analysis, the effects of changes in body mass were highly exceeded by the steroid effect which confirms the muscle-catabolic effect of steroids. Other authors identified acute GvHD, myeloablative conditioning, and a higher dose of infused stem cells as a risk factor for rapid bone loss at 1 year after HSCT, whereas chronic GvHD and steroid use were both unfavourable prognostic factors in terms of osteopenia or osteoporosis at 2 years [17]. 

Bisphosphonates are widely accepted for the treatment of osteoporosis and osteopenia. They have proven to be effective to increase BMD and to decrease the rate of skeletal complications in a variety of patient populations [10]. There are data provided from 4 small studies which investigated the use of reabsorptive treatments (mostly zoledronic acid) for the prevention or treatment of bone loss after allogeneic HSCT [14–17]. Despite considerable numbers of included patients (*n* = 12 up to 60 patients), ZA was not applied to all of them (*n* = 12 up to 18 patients), and moreover, except one, the observational period within these trials was restricted to 12 months [14–17]. In all of these studies ZA increased significantly both lumbar and femoral BMD. Moreover, hydroxyproline excretion in urine decreased during the observational period [14, 15]. These findings seem to be consistent with the present study. BMD and T-score measurements significantly increased during the study period of 2 years and deoxypyridinoline levels decreased within this time. One can therefore conclude that ZA not only reduces bone loss after allo-HSCT, but rather increases BMD even though when patients are already compromised with osteoporosis or osteopenia.

The increase in BMD was evident throughout the whole study population and during the whole study period. Nevertheless, there were some striking differences in BMD increase within subgroups. The percentual increase in BMD was significantly higher in patients at a younger age, in patients whose donor was a female and who had received immunosuppression with CSA/MTX. The latter may be speculatively explained by the finding that MMF is associated with hypocalcaemia and hypomagnesaemia in approximately 30%. But this had never been evaluated in a study.

In contrast to previous studies of Chae et al., and Schulte and Beelen, our data have not confirmed an influence of corticosteroid treatment on BMD increment [5, 17]. One explanation for this finding is that the subgroups were too small to detect such a difference. Analyzing those who had received steroids prior to study entry, one can suggest a trend towards an increased BMD during treatment with ZA (+8.2% previous steroids versus +5.5% no prior steroids). 

Certainly, there are limitations of the present study consisting of a low study number, loss of patients who had undergone BMD measurements according to the study protocol (26 out of 36 patients), inclusion of patients with osteopenia and osteoporosis, and, moreover, limitations consisting of the heterogeneity of an allotransplant patient population (e.g., underlying disease, conditioning protocol, TBI). All these transplantation-associated factors certainly contribute to the presented results.

Multiple factors contribute to bone loss after allo-HSCT. Long-term survivors are confronted with severe problems, if they develop osteopenia- or osteoporosis-related complications as chronic pain and/or fractures. In summary, ZA was found to be effective not only to prevent but also to treat evident bone loss in the femoral hip and spine of patients after allo-HSCT. Thus, patients who are at a high risk for bone loss should be monitored carefully and should be considered to use ZA to prevent and to treat bone loss and skeletal events. However, the optimal time of initiation and duration requires further studies.

## Figures and Tables

**Table 1:** Patient characteristics prior to study entry.

	All patients (*n* = 36)	BMD group (*n* = 26)
*Baseline*		
Median age (yrs) at HSCT (range)	43.8 (18–64)	43.9 (18–64)
Gender (male/female)	24/12	18/8
Underlying disease		
AML	15	11
CML	11	8
ALL	5	4
NHL	3	1
MDS	2	2

*Allo-HSCT*		
Type of HSCT (BM/PBSCT/both)	16/16/4	12/10/4
HLA (identical/different)	28/8	19/7
Donor (related/unrelated)	19/17	15/11
Donor sex (male/female)	18/18	12/14
Cytoreduction prior to conditioning	11	9
TBI (*n*/4–8 Gy/10–12 Gy)	9/12/15	7/9/10
Busulphan	9	7
Cyclophosphamide	35	26
ATG	32	23
Immunosuppression		
CSA-MTX	24	18
CSA-MMF	12	8

*Post-allo-HSCT*		
Acute/chronic GvHD	28/26	20/17
Corticosteroids prior to study	29	20
Duration (days) ± SD	152 ± 173	159 ± 198
Cumulative dose (g) ± SD	11.1 ± 8.6	11.1 ± 9.1
Corticosteroids during the study	29	20
Duration (days) ± SD	628 ± 209	625 ± 121
Cumulative dose (g) ± SD	5.4 ± 2.4	5.2 ± 1.6

**Table 2:** Percentual BMD increase (%) of femur hip-significant and nonsignificant parameters (BMD group *n* = 26).

		*n*	1 year	2 years	*P*	*P*

					1 year	2 years
Age	<45 years	16	8.9	12.0	0.007	0.02
	≥45 years	10	2.9	3.4		
Donor gender	Female	14	7.6	10.2	0.04	0.05
	Male	12	2.9	4.4		
Immunosuppression	CSA/MTX	18	7.1	9.3	0.01	0.02
	CSA/MMF	8	2.2	3.9		
Gender	Male	18	6.5	8.6	—	—
	Female	8	3.5	5.4	—	—
BMD	Osteopenia	13	5.5	7.8	—	—
	Osteoporosis	13	9.6	7.8	—	—
Prior steroids	Yes	20	5.9	8.2	—	—
	No	6	4.3	5.5	—	—
Steroids during study	Yes	20	5.3	8.8	—	—
	No	6	5.6	7.2	—	—
Diagnosis	CML	8	5.5	6.7	—	—
	AML	11	4.7	6.6	—	—

(—) Not significant.

**Table 3:** Metabolic parameters (mean values), *n* = 36.

		Baseline	1 year	2 years
Serum	Calcium (mmol/L)	2.37 ± 0.1	2.34 ± 0.1	2.43 ± 0.4
	Phosphate (mg/dL)	3.6 ± 0.7	2.97 ± 1.6	3.48 ± 0.9
	Creatinine (mg/dL)	1.06 ± 0.2	1.1 ± 0.3	1.12 ± 0.3
	Albumin (g/dL)	4.0 ± 0.43	4.3 ± 0.4	4.48 ± 0.48
Urine	Ca (mmol/24 h)	5.15 ± 2.3	3.3 ± 0.8	3.44 ± 3.0
	Dpd* (nmol/nmol creatinine)	6.2 ± 4.6	3.6 ± 3.2*	3.2 ± 1.4**
Hormones°	Estradiol (pg/mL)	20.3 ± 9.9	20.8 ± 6.0	24.4 ± 16.9
	FSH (IU/L)	43.9 ± 25.6	70.9 ± 46	43.7 ± 33.0
	LH (lU/L)	21.3 ± 16.2	31.6 ± 22.3	21.9 ± 18.5
Vitamin D3	OH (ng/mL)	10.4 ± 6.2	17.8 ± 11.7	26.7 ± 11.2
	1,25 di-OH (pg/mL)	30.6 ± 19.4	49.4 ± 15.0	45.3 ± 20.9

± SD: standard deviation; Dpd: deoxypyridinoline, *P = 0.009, **P = 0.044; °5/12 women had received hormone replacement therapy.
